# Data-Driven Sequence of Changes to Anatomical Brain Connectivity in Sporadic Alzheimer’s Disease

**DOI:** 10.3389/fneur.2017.00580

**Published:** 2017-11-07

**Authors:** Neil P. Oxtoby, Sara Garbarino, Nicholas C. Firth, Jason D. Warren, Jonathan M. Schott, Daniel C. Alexander, For the Alzheimer’s Disease Neuroimaging Initiative

**Affiliations:** ^1^Progression of Neurodegenerative Disease Group (POND), Centre for Medical Image Computing, Department of Computer Science, University College London, London, United Kingdom; ^2^Dementia Research Centre, Department of Neurodegenerative Disease, UCL Institute of Neurology, University College London, London, United Kingdom

**Keywords:** brain connectivity analysis, data-driven, Alzheimer’s disease, disease progression modeling, graph theory analysis, computational model

## Abstract

Model-based investigations of transneuronal spreading mechanisms in neurodegenerative diseases relate the pattern of pathology severity to the brain’s connectivity matrix, which reveals information about how pathology propagates through the connectivity network. Such network models typically use networks based on functional or structural connectivity in young and healthy individuals, and only end-stage patterns of pathology, thereby ignoring/excluding the effects of normal aging and disease progression. Here, we examine the sequence of changes in the elderly brain’s anatomical connectivity over the course of a neurodegenerative disease. We do this in a data-driven manner that is not dependent upon clinical disease stage, by using event-based disease progression modeling. Using data from the Alzheimer’s Disease Neuroimaging Initiative dataset, we sequence the progressive decline of anatomical connectivity, as quantified by graph-theory metrics, in the Alzheimer’s disease brain. Ours is the first single model to contribute to understanding all three of the nature, the location, and the sequence of changes to anatomical connectivity in the human brain due to Alzheimer’s disease. Our experimental results reveal new insights into Alzheimer’s disease: that degeneration of anatomical connectivity in the brain may be a viable, even early, biomarker and should be considered when studying such neurodegenerative diseases.

## 1 Introduction

There is a growing body of literature using advanced computational and statistical modeling to understand progressive disorders. The majority of these has been applied to the most common neurodegenerative disorder of the brain, Alzheimer’s disease (AD)—see Ref. (1), for a review of the field. Discriminative models range from simple diagnosis using supervised machine-learning classifiers (2–4) to unsupervised clustering of disease subtypes (5–7). Such discriminative models typically do not directly include a notion of disease progression. By contrast, generative data-driven models (8–11) estimate disease progression signatures that can aid disease understanding from start to end. Such an understanding is vital for the early identification of individuals who are likely to be responsive to a particular therapy, and also for identifying the earliest pathological changes for designing such therapies. The protracted duration of AD, and especially the decades-long presymptomatic phase, makes this a challenging disorder to study in the general population. Dominantly inherited variants of AD, e.g., Ref. (12)., can be used to alleviate this problem to some extent, but the open question of whether dominantly inherited AD is a suitable model for sporadic late-onset AD remains unanswered.

Alzheimer’s disease has been widely described as a disconnection syndrome (13–16). This has contributed to the motivation behind the relatively recent emergence of network models of neurodegeneration. The motivation is to understand mechanisms of disease propagation by relating models of brain networks to observed pathology. The networks are abstract representations of “connections” between brain regions and can be constructed from (1) functional correlations during rest or activity (usually estimated using blood-oxygen-level-dependent contrast MRI (17)), (2) gray-matter covariance (estimated from structural MRI), or (3) anatomical connections (estimated using neuronal tractography from diffusion-weighted MRI).

Network spreading models attempt to explain neurodegeneration in terms of the transneuronal spread of prion-like (18) pathogens such as abnormal proteins. Anatomical connectivity networks are a natural choice for such models as they estimate physical connections between brain regions, rather than the correlations estimated in functional and gray-matter structural covariance networks. Previous work in network spreading models of neurodegenerative diseases has considered how well these models can predict end-stage disease by: correlating patterns of healthy intrinsic (functional) connectivity and gray-matter volume (14); using healthy functional connectivity to compare network-based mechanistic hypotheses of AD progression (19); and using healthy anatomical connectivity to predict atrophy (20), amyloid load (21), or metabolism (22). All of these network spreading models used *static* connectivity of healthy, *young* individuals to build networks with which to predict end-stage AD pathology. This ignores the effects of aging and disease progression on the network substrate being used to predict pathology.

In contrast to previous network spreading models, we consider *elderly* connectivity networks, and we explicitly model the course of disease progression. This enables us to investigate how the brain’s anatomical connectivity *changes* with AD progression. We do this in a novel manner by analyzing regional network (graph-theoretic (23, 24)) measures of brain connectivity in groups of healthy and diseased individuals, within the context of an event-based model (8, 10) of disease progression. The model produces a data-driven, fine-grained signature of the sequence of disease-related changes in anatomical connectivity of the human brain, including uncertainty in the sequence. Our innovations beyond earlier event-based models (8, 10) include analyzing biomarkers of brain connectivity and employing a new nonparametric mixture modeling technique (25) for estimating biomarker abnormality that is built upon kernel density estimation (26, 27).

## 2 Materials and Methods

**Figure 1:**
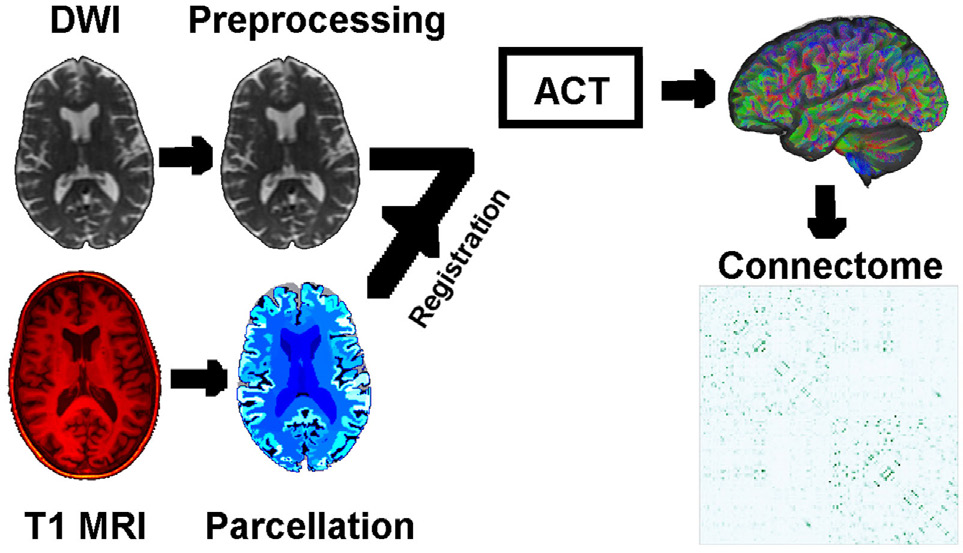
Schematic of the image analysis pipeline, per individual: DWI preprocessing → DWI normalization → rigid registration of T1 MRI to DWI → T1 parcelation → anatomically constrained tractography → connectomes based on density of WM neuronal connections between GM regions of interest. Abbreviations: DWI, diffusion-weighted image; MRI, magnetic resonance image; ACT, anatomically constrained tractography; WM/GM, white/gray matter.

Our analysis can be summarized as three steps. First, we used probabilistic anatomically constrained tractography to construct individual whole-brain connectomes for imaging data from 168 participants from the Alzheimer’s Disease Neuroimaging Initiative (ADNI) study, across four age-matched and education-matched clinical categories (4 × 42 per category, the maximum available in one of the categories). The image analysis pipeline is visualized in the schematic of Figure 1. Second, from each connectome, we computed various local network measures (graph-theory metrics) representing the topology of anatomical connectivity in regions of each participant’s brain. Finally, we estimated the ordered sequence in which these measures become abnormal using an event-based model (8, 10).

### 2.1 Data

Data used in the preparation of this article were obtained from the Alzheimer’s Disease Neuroimaging Initiative (ADNI) database.^1^ The ADNI was launched in 2003 as a public–private partnership, led by Principal Investigator Michael W. Weiner, M.D. The primary goal of ADNI has been to test whether serial magnetic resonance imaging (MRI), positron emission tomography (PET), other biological markers, and clinical and neuropsychological assessment can be combined to measure the progression of mild cognitive impairment (MCI) and early Alzheimer’s disease (AD).

In February 2017, we remotely accessed the Laboratory of NeuroImaging’s Image Data Archive at the University of Southern California and searched for suitable participants to include in our anatomical connectome cohort: ADNI participants whose brains were imaged with both structural magnetic resonance imaging (MRI) and diffusion-weighted imaging (DWI) at a single study visit. We sought age-matched groups across the four diagnoses of Cognitively Normal (CN), Early Mild Cognitive Impairment (EMCI), Late Mild Cognitive Impairment (LMCI), and probable AD (AD). This resulted in 42 participants per group. We downloaded the unprocessed structural MRI (3.0T, T1-weighted, non-accelerated IR-SPGR; GE Medical Systems) and DWI (Axial diffusion tensor imaging; GE Medical Systems) for the first available suitable visit of these 168 participants from the ADNI2 phase of ADNI (including: 5 rollovers from the first ADNI phase, ADNI1; and a single rollover from the ADNI Grand Opportunity phase, ADNIGO). Herein, we refer to our anatomical connectome cohort as *The168*. We also downloaded associated demographics data and metadata in CSV format. Demographics for *The168* are summarized in Table 1, and for all ADNI2 (at ADNI2 baseline) in Table 2.

**Table 1:** Demographics by diagnosis for our anatomical connectome cohort, *The168*: 168 ADNI2 participants included in this study.

	CN	EMCI	LMCI	AD
*n* (Females)	42 (23)	42 (17)	42 (16)	42 (17)
*n* APOE+ (APOE−)	14 (27)	20 (21)	31 (10)	28 (13)
*n* AV45+ (AV45−)	15 (**26**)	22 (17)	29 (6)	**38** (4)
Age mean (SD)	73 (6)	73 (7)	73 (7)	73 (7)
Edu mean (SD)	16 (3)	16 (3)	16 (3)	16 (3)

*One-way ANOVA implies that the groups do not differ significantly by age (*p* > 0.97), nor education (*p* > 0.45). Amyloid positivity was set at cortical mean AV45 SUVR ≥ 1.10 (28), prior to adjustment for covariates. Numbers in bold font indicate controls and patients (see text for details) used in our disease progression modeling*.

**Table 2:** Demographics by diagnosis for all ADNI2 participants at initial visit.

	CN	EMCI	LMCI	AD
*n* (Females)	336 (174)	277 (124)	450 (183)	276 (121)
*n* APOE+ (APOE−)	94 (240)	116 (157)	236 (212)	184 (89)
*n* AV45+ (AV45−)	52 (**106**)	105 (100)	98 (42)	**122** (15)
Age mean (SD)	75 (6)	71 (7)	74 (7)	75 (8)
Edu mean (SD)	16 (3)	16 (3)	16 (3)	15 (3)

*As in Table 1, amyloid positivity was set at cortical mean AV45 SUVR ≥ 1.10 (28), prior to adjustment for covariates. Numbers in bold font indicate controls and patients (see text for details) used in our disease progression modeling*.

Our disease progression modeling requires both a control and a patient group, which we defined as amyloid-negative CN participants and amyloid-positive AD participants, respectively (see bold figures in Table 1). The threshold for amyloid positivity was chosen as an amyloid PET (Florbetapir ^18^F-AV-45, hereafter AV45) cut-point from the literature (28): AV45 Standardized Uptake Value Ratio *SUVR* ≥ 1.10, which was based on the upper 95% confidence interval for young healthy subjects. Using this criteria, in *The168*, we identified 26 controls and 38 patients out of a possible maximum of 42 each. For the wider ADNI2 cohort (including rollovers from ADNI1 and ADNIGO), we found 106 controls and 122 patients—see the bold figures in Table 2.

### 2.2 Connectomics

Structural connectomes were generated using tools provided in the MRtrix3 software package,^2^ customized to work with the Geodesic Information Flows algorithm (29) for segmentation and parcelation. The pipeline included DWI denoising (30), preprocessing (31, 32), and bias field correction (33); inter-modal registration (34); T1 tissue segmentation (29); spherical deconvolution (35, 36); probabilistic tractography (37) utilizing anatomically constrained tractography (38) and dynamic seeding (39); spherical deconvolution informed filtering of tractograms (SIFT) (40); T1 parcelation (29); and robust structural connectome construction (41). We used the dwiintensitynorm script in MRtrix3 during DWI preprocessing, but found it necessary to modify the subsequent usage of the population_template script such that DWI masks were not used to create the template (the DWI were pre-masked). Note that we use SIFT to produce biologically plausible tractograms where the streamline density in each voxel matches the fiber orientation distributions estimated from the DWI, instead of simply thresholding the number of fibers connecting two regions.

Our anatomical connectome for each participant is a weighted adjacency matrix that includes only inter-node connections across 130 regions of interest consisting of cortical and subcortical gray-matter regions (including striatal), plus the cerebellum and brain stem. Weights, or connection strengths, are normalized to [0, 1], and so represent within-participant anatomical connection density. The image analysis pipeline is visualized in Figure 1. The 130 regions of interest are a subset of those in the labeling protocol used by Geodesic Information Flows (29, 42), which is a modified version of the Desikan–Killiany–Tourville protocol (43).

### 2.3 Anatomical Connectivity Metrics

For each participant and region of interest, we calculated 12 brain connectivity metrics from the anatomical connectomes, using the Brain Connectivity Toolbox (24) in MATLAB, after appropriate normalization using the weight_conversion function. The local network metrics fall into the following broad categories:

1. Hubs (basic centrality): degree, strength, degree z-score;
2. Importance (advanced centrality and shortest paths): efficiency, characteristic path length, participation coefficient, betweenness centrality, eigenvector centrality, PageRank centrality (44);
3. Segregation/integration: clustering coefficient, eccentricity.

For each region of interest, the medians of the controls and patients distributions for each network measure were statistically compared using a Mann–Whitney–Wilcoxon rank-sum test. Only measures where *p* < 0.05/12 (Bonferroni-corrected within region) were retained for further analysis within the event-based model of disease progression—we refer to such biomarkers as having “disease signal.”

Note that local efficiency and local clustering coefficient return similar information to each other, as do eigenvector centrality and PageRank centrality, and so one may ask whether we are including redundant information in our models. We argue that we are not, as seen in our results where: (1) local efficiency contained disease signal, whereas local clustering coefficient did not and (2) the centrality measures appear in different positions within the model sequences.

### 2.4 Event-Based Model of Disease Progression

The event-based model (EBM) (8, 10) is a data-driven approach for probabilistically sequencing a cross-sectional set of scalar measurements (“biomarkers”) in the order in which they become observably abnormal. In this context, an “event” constitutes a biomarker appearing more abnormal/diseased than normal/healthy. The EBM is able to estimate an average sequence of disease progression events from cross-sectional data because the proportion of abnormal measurements within a cohort will decline in concert with the average ordering. That is, the biomarker that changes earliest (the first disease event) will contain the highest proportion of abnormal measurements (from affected and presymptomatic individuals), and so on. The EBM fuses multiple biomarker measurements across individuals, with the simplest versions assuming a single disease progression sequence for all individuals, as done here. Determining biomarker abnormality in a data-driven manner necessitates mixture modeling within biomarkers, for which we use a new method (25), described below in Section 2.4.1. For convenience, we normalize all biomarkers to “c-scores” (standardized z-scores relative to controls) that increase with abnormality. Otherwise, we used the same fitting procedures as in Ref. (10). Cross-validation of our EBMs was estimated by refitting the sequence (but not the event measures) to 100 separate bootstrap samples from the data.

We planned to build four EBMs of network connectivity changes, corresponding to the three broad categories of connectivity in Section 2.3, plus all biomarkers together:

- “EBM0”—non-network biomarkers.
- “EBM1”—EBM0 markers, plus biomarkers of anatomical brain network hubs.
- “EBM2”—EBM0 markers, plus biomarkers of anatomical brain network importance: centrality and shortest paths.
- “EBM3”—EBM0 markers, plus biomarkers of segregation/integration in the anatomical brain network.
- “EBM4”—all biomarkers.

EBM0 acts as a reference point and for investigating consistency with previous EBMs of AD (10, 45) and includes only non-network biomarkers: average cortical level of amyloid (from AV45 PET) and hypometabolism (from fludeoxyglucose (FDG) PET), test score on the Mini-Mental State Examination (MMSE) (46), and structural MRI volumes of the hippocampus, entorhinal area, ventricles, and whole brain. Biomarkers were adjusted for healthy age, education, and gender using regression (residuals method, controls only). Brain volumes were also adjusted for intracranial volume. We did not fit EBM3 because there was no disease signal (see Section 2.3) in regional clustering coefficients nor eccentricity.

#### 2.4.1 Biomarker Event Measures

The probability of a biomarker event is fundamental for sequencing the biomarker events into a pathological cascade of disease progression. Since not all patients will have experienced later events, and indeed some controls will have already experienced the earliest events in the cascade, it is necessary to fit a mixture model in order to discover the event probability. Previous EBM analyses (8, 10, 45, 47–49) used mixtures of parametric probability distributions such as Gaussian and uniform. Here, we use a new method (25) that fits a mixture of nonparametric kernel density estimate (KDE) distributions.

## 3 Results

### 3.1 Global Connectivity

**Figure 2:**
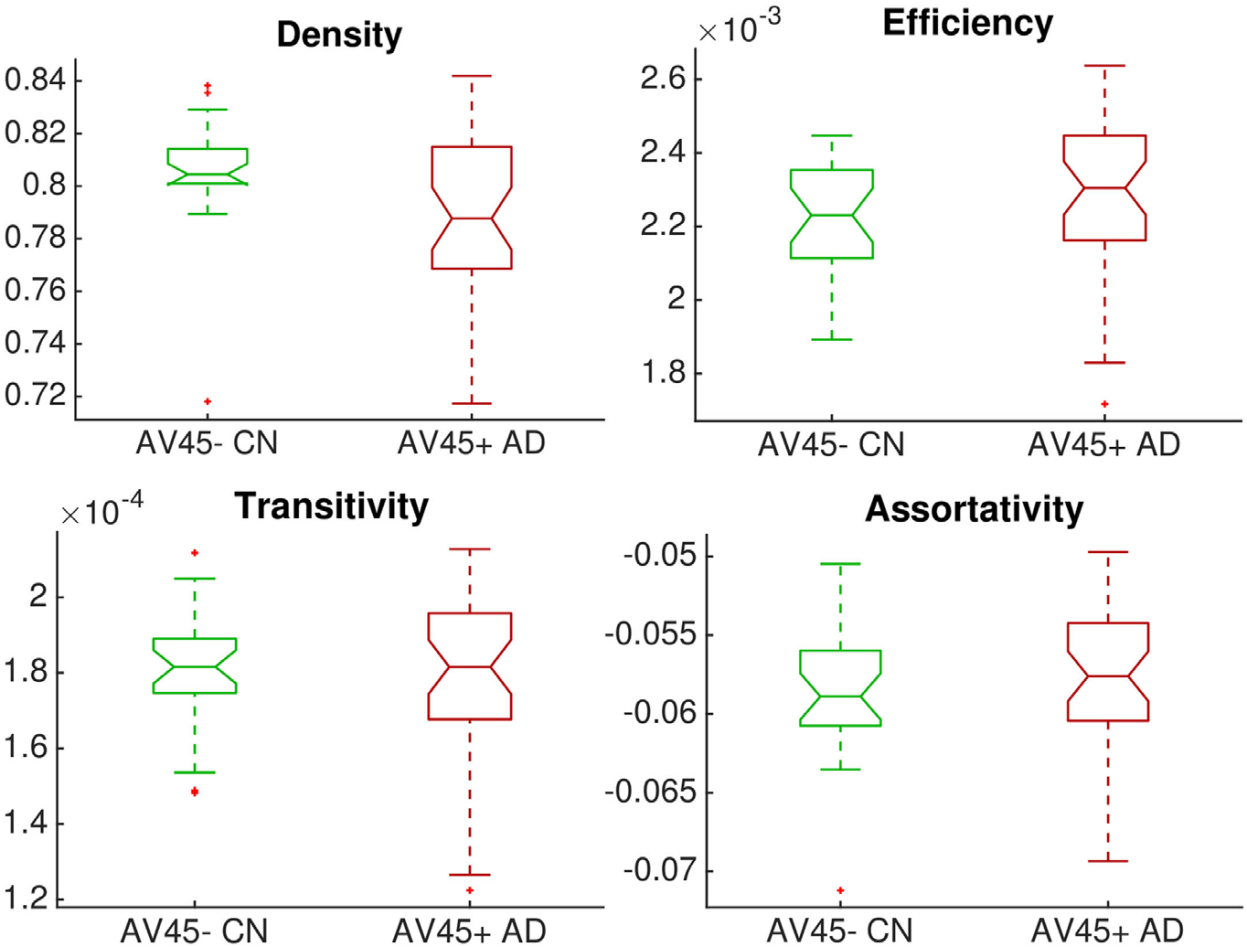
Group comparison of dimensionless global network measures between controls (amyloid-negative CN, green) and patients (amyloid-positive probable AD, red). There were no significant differences between groups.

Figure 2 shows that global network connectivity in health and disease did not differ significantly in our cohort, *The168*. That is, we found no significant group-level difference between the 26 controls (AV45-positive CN) and 38 patients (AV45-negative AD) across four global brain network metrics: density (connectedness), efficiency (segregation), transitivity (segregation), and assortativity (resilience). The null hypothesis in each Mann–Whitney–Wilcoxon rank-sum test was accepted using a Bonferroni-corrected significance level of *p* = 0.05/4 = 0.0125: density *p* = 0.0147; transitivity *p* = 1; efficiency *p* = 0.145; assortativity *p* = 0.363.

### 3.2 Event-Based Models

#### 3.2.1 Biomarkers

**Figure 3:**
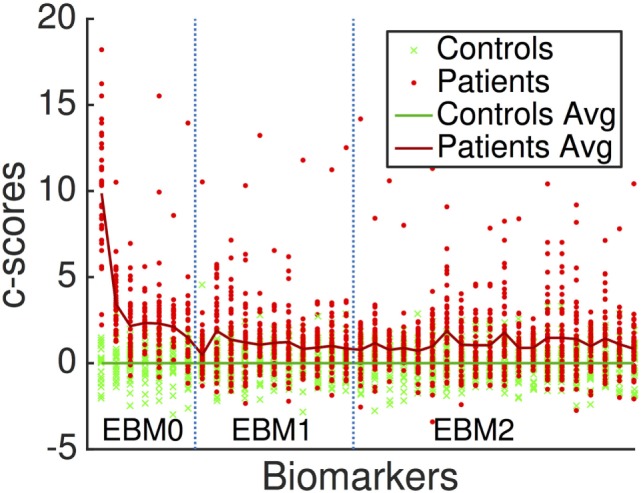
Standardized “c-scores” for all biomarkers included in our analyses. Non-network markers are to the left, denoted EBM0. Network metrics are to the right, delineated by EBM1 (hubs, i.e., basic measures of centrality) and EBM2 (importance, i.e., advanced measures of centrality and shortest paths). EBM3 was not generated since no segregation/integration-based network measures contained disease signal. EBM4 includes all biomarkers.

Figure 3 is a visualization of disease signal in the full set of 38 biomarkers included in the EBMs. The vertical axis is the standardized “c-score” for each biomarker along the horizontal axis: c-score is biomarker value standardized to controls (c.f., z-scores). Group-average lines are shown with individual data points as green crosses for controls and red dots for patients.

#### 3.2.2 Disease Progression Sequences

The EBM estimates a data-driven probabilistic sequence of biomarker abnormality, which we visualize as plots of grayscale positional variance (horizontal axis) around the maximum-likelihood ordering (vertical axis). The strongest possible ranking of biomarker abnormality would appear as a black diagonal.

**Figure 4:**
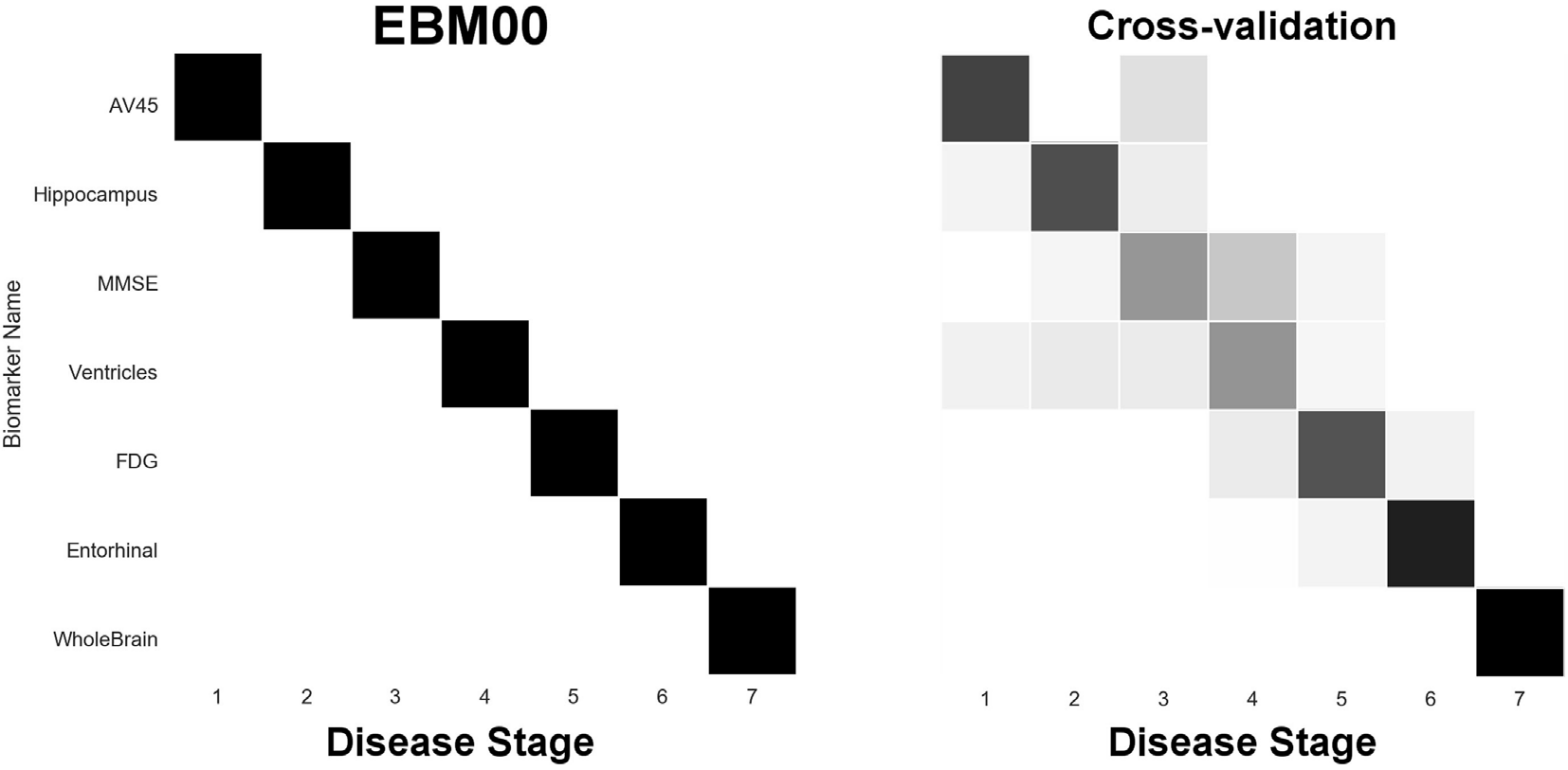
EBM for selected standard (non-network) biomarkers, built on available ADNI2 baseline data. Left panel: left-right positional variance of the maximum-likelihood sequence. Right panel: cross-validation of the sequence from bootstrapping.

Our first experiment was to build “EBM00”—an EBM including only non-network biomarkers, and using available ADNI2 baseline data (summarized in Table 2). The results are shown in Figure 4, with positional variance estimated from the MCMC fitting procedure (8) shown in the left of the figure, and from bootstrapping shown in the right of the figure (cross-validation). The EBM00 sequence is consistent with current understanding of AD progression (and previous EBMs (10)): early amyloidosis and hippocampal volume loss, followed by cognitive decline, then hypometabolism and broader neurodegeneration.

**Figure 5:**
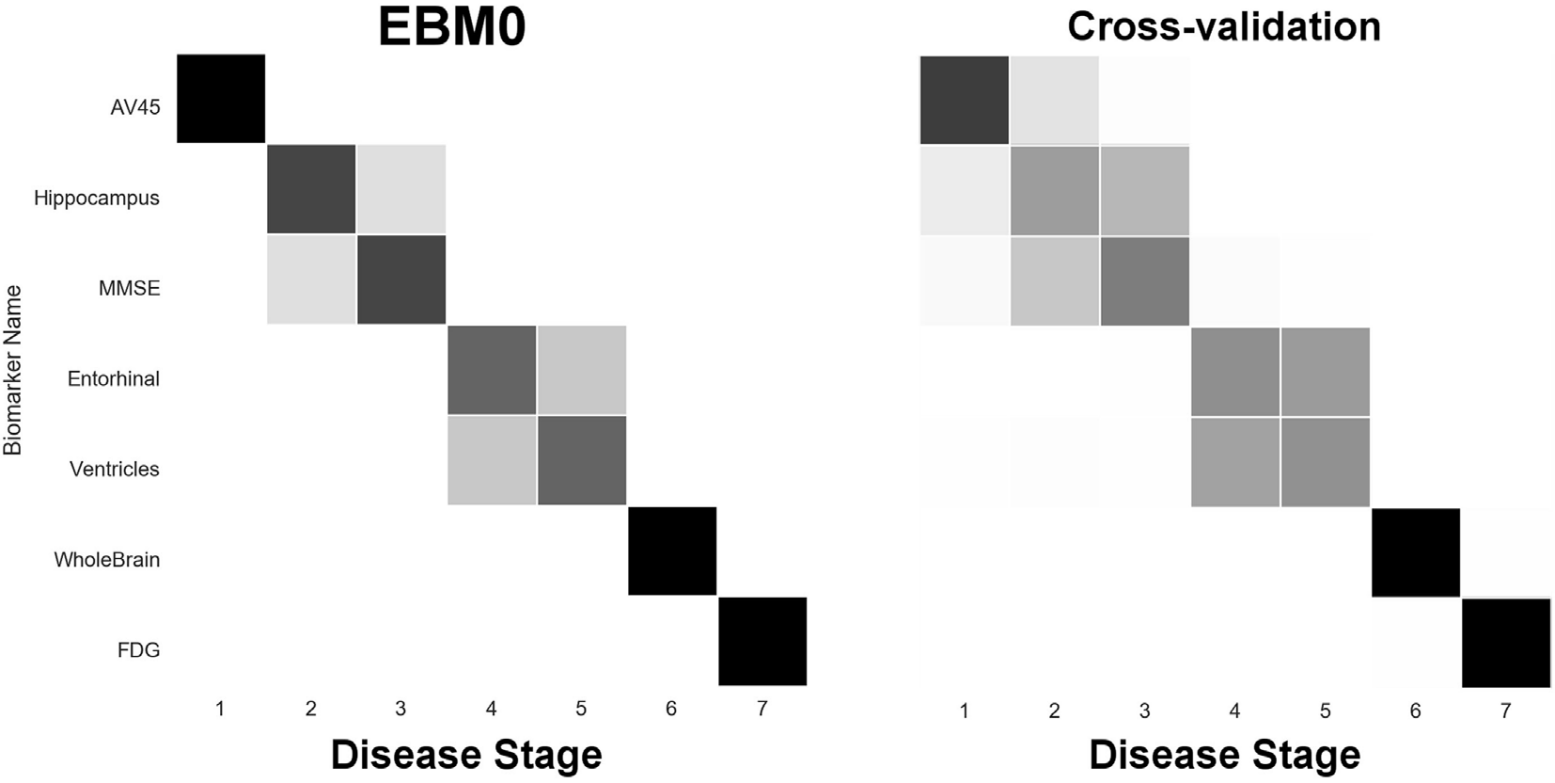
EBM for selected standard (non-network) biomarkers, built on data from our anatomical connectome cohort, *The168*. Left panel: left-right positional variance of the maximum-likelihood sequence. Right panel: cross-validation of the sequence from bootstrapping.

In our next experiment, we built “EBM0”—the same biomarkers as EBM00, but using only data from our anatomical connectome cohort, *The168* (Table 1). The results are shown in Figure 5, with the positional variance diagram from fitting on the left, and from bootstrapping on the right. Like EBM00, the EBM0 sequence is consistent with current understanding of AD progression, with only one exception: the apparent late appearance of hypometabolism (FDG). We attribute this to the relatively small number of probable AD patients in *The168* with abnormally high metabolism, compared to the full ADNI2 cohort (Figure S1 in Supplementary Material). We also note that the positional variance is larger in EBM0 than in EBM00, probably due to the lower numbers of individuals.

**Figure 6:**
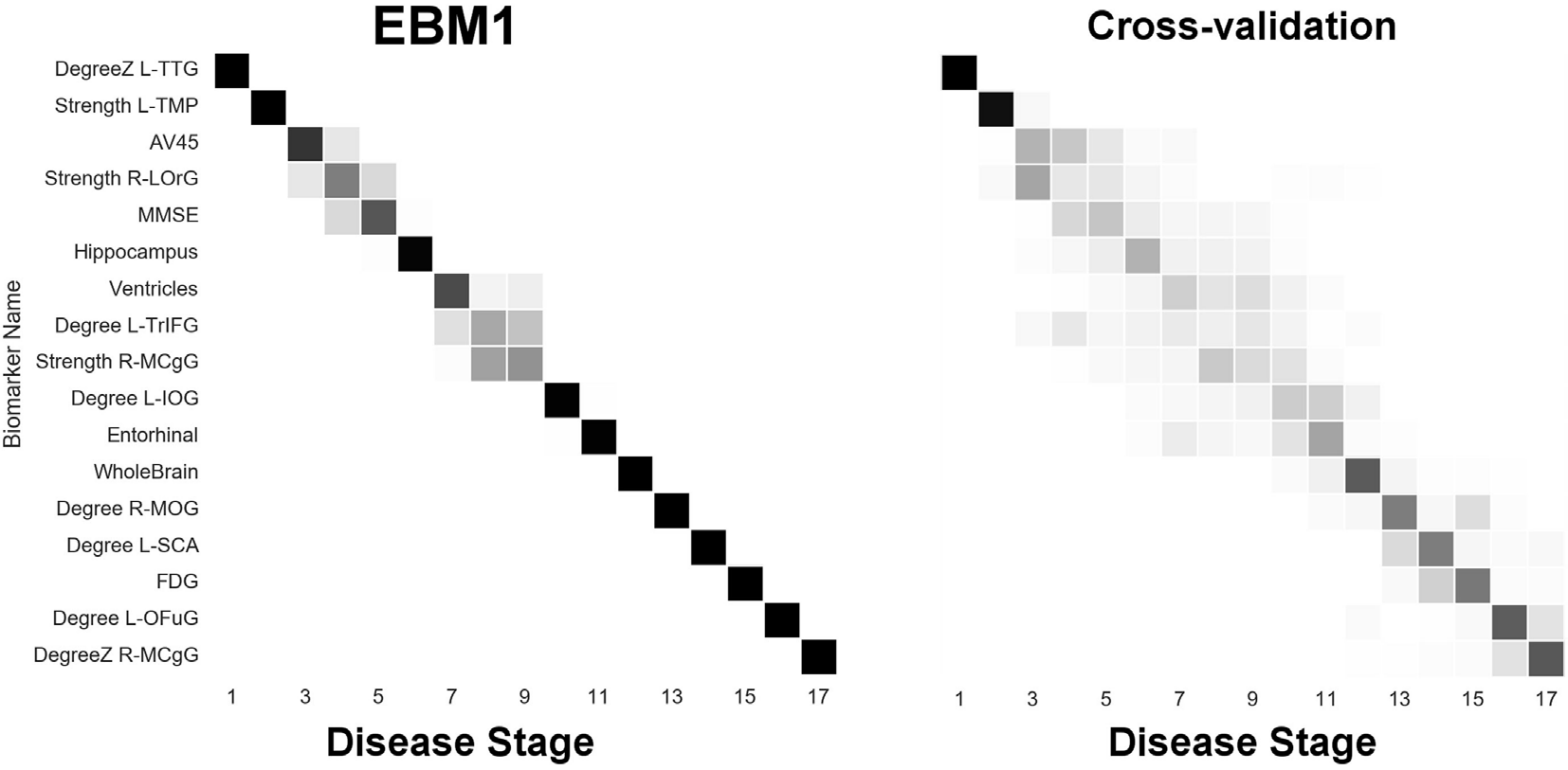
EBM for hubs in the brain’s anatomical network, along with selected standard (non-network) biomarkers, built on data from our anatomical connectome cohort, *The168*. Left panel: left-right positional variance of the maximum-likelihood sequence. Right panel: cross-validation of the sequence from bootstrapping. Abbreviations: DegreeZ, degree z-score; L, left; R, right; TTG, transtemporal gyrus; TMP, temporal pole; LOrG, lateral orbital gyrus; TrIFG, triangular inferior frontal gyrus; MCgG, middle cingulate gyrus; IOG, inferior occipital gyrus; MOG, middle occipital gyrus; SCA, subcallosal area; OFuG, occipital fusiform gyrus.

**Figure 7:**
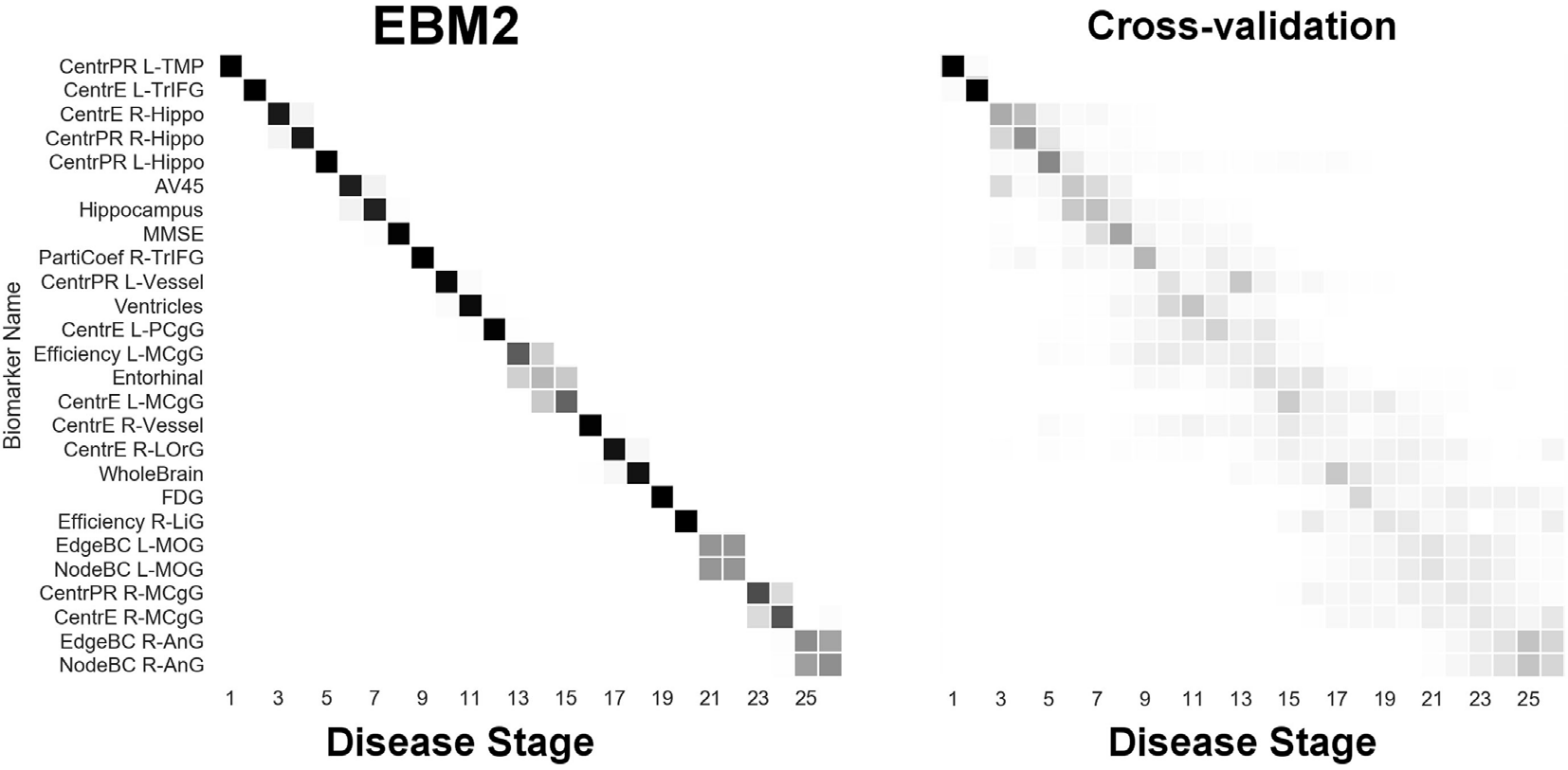
EBM for regional centrality in the brain’s anatomical network, along with selected standard (non-network) biomarkers, built on data from our anatomical connectome cohort, *The168*. Left panel: left-right positional variance of the maximum-likelihood sequence. Right panel: cross-validation of the sequence from bootstrapping. Abbreviations: CentrPR, PageRank centrality; CentrE, eigenvector centrality; PartiCoef, participation coefficient; EdgeBC, edge betweenness centrality; NodeBC, node betweenness centrality; L, left; R, right; TMP, temporal pole; TrIFG, triangular inferior frontal gyrus; Hippo, hippocampus; PCgG, posterior cingulate gyrus; MCgG, middle cingulate gyrus; LOrG, lateral orbital gyrus; LiG, lingual gyrus; MOG, middle occipital gyrus; AnG, angular gyrus.

**Figure 8:**
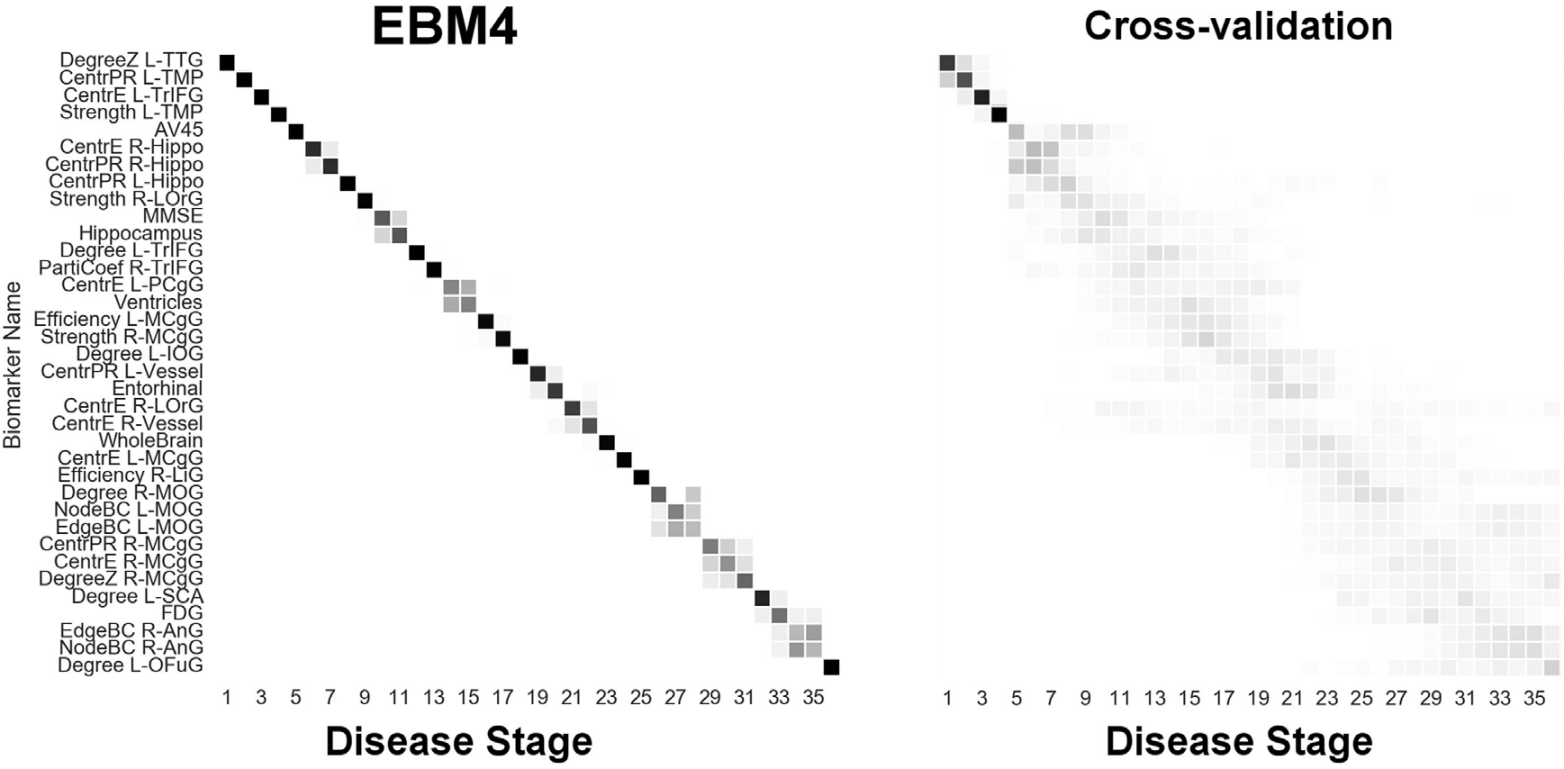
EBM for all biomarkers considered, including hubs and centrality in the brain’s anatomical network, along with selected standard (non-network) biomarkers, built on data from our anatomical connectome cohort, *The168*. Left panel: left-right positional variance of the maximum-likelihood sequence. Right panel: cross-validation of the sequence from bootstrapping. Abbreviations as in Figures 6 and 7.

Next, we report results from experiments on brain connectivity changes in AD. Figure 6 shows “EBM1,” which includes network biomarkers that measure hubs in the brain network through node degree or strength. Figure 7 shows “EBM2,” which includes biomarkers of network importance as measured by centrality and shortest path metrics. Figure 8 is “EBM4” which includes both hubs and centrality. (“EBM3” does not exist because no biomarkers of segregation/integration passed the test for disease signal—see Section 2.3.). The network biomarker event measures are presented in Figure S2 in the Supplementary Material.

From Figure 6 we can infer the ordering the network biomarker event measures (from mixture modeling) are presented in Figure S2 in the Supplementary Material. In which certain anatomical network hubs of the brain deteriorate. We note early involvement of regions in the temporal lobe: the left transverse temporal gyrus (TTG; a.k.a. Heschl’s gyrus; auditory cortex) and the left temporal pole (TMP; anterior of the temporal lobe); and a region in the frontal lobe: the right lateral orbital gyrus (LOrG). The model suggests that the later hubs to deteriorate include the middle occipital gyrus (MOG), subcallosal area (SCA), left occipital fusiform gyrus (OFuG), and right middle cingulate gyrus (MCgG). Many, but not all, of these regions are involved in the default mode network (DMN), see, e.g., Ref. (50), and references within.

From Figure 7 we infer the sequential deterioration of regional importance in the anatomical network of the brain, as measured by local centrality and efficiency. The model suggests that centrality declines first in memory-related parts of the DMN in the temporal and prefrontal lobes: left temporal pole (TMP), left triangular part of the inferior frontal gyrus (TrIFG), bilateral hippocampus; and last in regions from the occipital (and anterior limbic) lobe(s): right lingual gyrus (LiG; visual cortex), left middle occipital gyrus (MOG), right middle cingulate gyrus (MCgG), and right angular gyrus (AnG).

Finally, Figure 8 (“EBM4”) contains all biomarkers of anatomical connectivity included in this study. The ordering of abnormality in hubs and centrality biomarkers is consistent with EBM1 and EBM2, respectively. Figure 8 enables comparison of the relative sequential decline among hubs and regional centrality in the AD brain’s anatomical connectivity network. We observe that the clearest and earliest decline in anatomical connectivity in the AD brain is both hubs and centrality in the left temporal lobe, all of which are DMN regions known to have memory-related storage and functions. Memory dysfunction is the clinical phenotype of typical AD dementia.

## 4 Discussion

### 4.1 Findings in Context

Brain network hubs are thought to experience increased susceptibility to AD pathology due to higher activity and metabolism, such as regions within the DMN, e.g., Ref. (50), and references within. Our experimental results are consistent with this idea, suggesting that the earliest abnormality in AD occurs in hubs (Figure 6) and other centrally important regions (Figure 7) in the left temporal lobe, and bilaterally in the hippocampus—see Figure 8. Specific left temporal lobe regions include the transverse temporal gyrus (part of the auditory cortex), the temporal pole (which may be involved in social and emotional cognition, e.g., because it has been shown to be affected in frontotemporal dementia (51)), and the triangular part of the inferior frontal gyrus (which may be involved in semantic memory (52)).

Our finding of early connectivity changes involving the auditory cortex is potentially of particular clinical relevance to Alzheimer’s disease: hearing impairment has recently emerged as a major risk factor for cognitive decline and a focus of intense epidemiological interest (53), while functional alterations in central auditory processing have been identified in both presymptomatic and established Alzheimer’s disease (54, 55). Our findings suggest how such alterations may fit within the pathogenic cascade of Alzheimer’s evolution.

Our results also suggest that this deterioration of anatomical connectivity in the brain network may be detectable prior to bulk estimates of amyloidosis, such as the mean SUVR across the cortex in AV45 PET. However, prior to claiming that network-based biomarkers may form sensitive early biomarkers of AD, we would seek confirmation using larger anatomical connectivity cohorts (see future work in Section 4.3).

Our experiments also identified that the anatomical connectivity of some DMN regions is not affected until later in the pathological cascade of AD. These included hubs in the angular gyrus, and hubs and centrality in the medial cingulate gyrus—see Figure 8.

We note that the ordering of brain regions involved in our sequence of anatomical connectivity changes to the Alzheimer’s disease brain (Figure 8) does not follow the ordering of regions in the neuropathological sequence identified by Braak and Braak (56). This suggests that changes in anatomical connectivity may not occur in sync with deposition of abnormal protein in brain tissue. Indeed, our results suggest that connectivity changes may appear before bulk deposition of amyloid across the cortex is detectable *in vivo*.

The brain’s anatomical connectivity network is altered by AD. These disease-related connectivity changes may be due to neurodegeneration in gray matter, alterations in deep white matter, or alterations in superficial white matter (57) located near the gray-matter/white-matter boundary. We designed our pipeline to be sensitive to these disease-related alterations by employing anatomically constrained tractography (38) and the SIFT method (40), which should improve the biological accuracy of the tractography-based connectivity estimates.

We have studied the late onset, typical variant of Alzheimer’s disease. It will be of interest to apply our technique to explore genetic factors that might influence the progression of Alzheimer’s disease; and to young onset Alzheimer’s disease where different phenotypic presentations are more commonly seen.

### 4.2 Novelty of This Work

Our approach differs from previous network spreading models in two very important ways. We investigated *dynamic* pathological disruption of the *elderly* brain’s anatomical connectivity network as a function of AD *progression*. Previous network models of AD (14, 19–22) used *static* connectivity patterns estimated from young and healthy individuals, then employed only end-stage patterns of pathology, such as atrophy, to study network spreading mechanisms. Thus, the previous approaches ignore both the effects of normal aging and the effect of disease progression on the network substrate being used to predict pathology.

Our approach provides utility beyond contributing to our understanding of the AD pathological cascade. These include the ability to estimate a longitudinal disease progression signature that is useful for patient staging (10), and for identification of candidate biomarkers for early diagnosis.

In this respect, we believe that our work may be the first data-driven quantification of the widely held idea that AD progression reflects specific anatomical network disruption, not just neurodegeneration.

### 4.3 Future Work

Future work will include seeking a separate, larger anatomical connectivity cohort upon which to validate our results. While our own cross-validation experiments reported above provide some level of confidence in our conclusions, external validation on a larger cohort would be all the more convincing. Validation on animal models of AD is a related idea that might be worth pursuing.

Our analyses could be applied to other neurodegenerative diseases. We will actively seek suitable cohorts from other diseases upon which to examine pathological changes in the connectivity of the human brain. This would subsequently extend quite naturally to include application of the models to differential diagnosis, and to the related problem of within-disease subtyping (7, 58).

On the highest level, our work involved comparison of graphs (59). There are subtle challenges to comparing graphs and metrics derived from them, even when the number of nodes is constant and the average number of connections is the same (60), as was the case in our study. In the future, we will consider normalizing our connectivity metrics with those derived from random graphs or by looking at distances between graphs (60).

### 4.4 Conclusion

We have sequenced the progressive deterioration of anatomical connectivity in the Alzheimer’s disease brain. We believe that this is the first attempt to do so in a data-driven manner that incorporates the effects of both aging and disease progression. Aging is accounted for by using healthy elderly brains as controls. Disease progression is estimated using event-based modeling.

Our experimental results reveal new insights into Alzheimer’s disease progression: that degeneration of anatomical connectivity in the brain may be a viable, even early, biomarker and should be considered when studying such neurodegenerative diseases.

## Author Note

Data used in preparation of this article were obtained from the Alzheimer’s Disease Neuroimaging Initiative (ADNI) database (http://adni.loni.usc.edu). As such, the investigators within the ADNI contributed to the design and implementation of ADNI and/or provided data but did not participate in analysis or writing of this report. A complete listing of ADNI investigators can be found at: http://adni.loni.usc.edu/wp-content/uploads/how_to_apply/ADNI_Acknowledgement_List.pdf.

## Author Contributions

NO, SG, and DA conceived the study and designed the experiments, in consultation with JS. NO performed the image analysis that produced the connectomes and regional volumes and wrote the manuscript. NO and SG collated the data and performed the event-based modeling analyses with input from NF (in particular the mixture modeling for event measures), JS, and DA. All authors contributed to interpretation of the results and the final version of the manuscript.

## Conflict of Interest Statement

The authors declare that the research was conducted in the absence of any commercial or financial relationships that could be construed as a potential conflict of interest.

## Footnotes

^1^http://adni.loni.usc.edu.

^2^http://mrtrix.org.
