# Supplementary material for: Data-Driven Sequence of Changes to Anatomical Brain Connectivity in Sporadic Alzheimer’s Disease
Source: Front Neurol. 2017 Nov 7;8:580. doi: 10.3389/fneur.2017.00580 (PMC5681907; doi:10.3389/fneur.2017.00580)
Supplement: Supplementary file 1 [file presentation_1.pdf]

Supplementary Material: Data Driven Sequence of Changes to Anatomical Brain Connectivity in Sporadic Alzheimer's Disease

Neil P. Oxtoby,[1,*] Sara Garbarino,[1] Nicholas C. Firth,[1,2] Jason D. Warren,[2] Jonathan M. Schott,[2] and Daniel C. Alexander,[1] for the Alzheimer's Disease Neuroimaging Initiative[**]

*Correspondence:
Neil P. Oxtoby
Centre for Medical Image Computing, Dept. of Computer Science, University College London, Gower Street, London WC1E 6BT, United Kingdom
n.oxtoby@ucl.ac.uk

**Data used in preparation of this article were obtained from the Alzheimer's Disease Neuroimaging Initiative (ADNI) database (`adni.loni.usc.edu`). As such, the investigators within the ADNI contributed to the design and implementation of ADNI and/or provided data but did not participate in analysis or writing of this report. A complete listing of ADNI investigators can be found at: `http://adni.loni.usc.edu/wp-content/uploads/how_to_apply/ADNI_Acknowledgement_List.pdf`.

1 SUPPLEMENTARY FIGURES

Within the event-based model (EBM), the probability of a biomarker event (biomarker event measure) is determined by a mixture model including patients and controls. In this work we used a novel mixture of Kernel Density Estimates (Firth, et al., Unpublished), for which a full manuscript is in preparation.

Figure S1 shows the mixture model components and biomarker event measures for the non-network biomarkers included in EBM00 (left, larger group derived from all ADNIGO/2) and EBM0 (right, smaller group derived from *The168* only), which highlights the similarities, and subtle differences, in biomarker distributions between the larger and smaller groups. In particular, the event probability (black line) for FDG appears earlier in the full cohort, with an inflection point at a c-score of ≈ 1 vs ≈ 2.5.

Figure S2 shows the mixture model components and event probabilities for the non-network and network biomarkers included in EBM1 (hubs) and EBM2 (centrality and shortest paths).

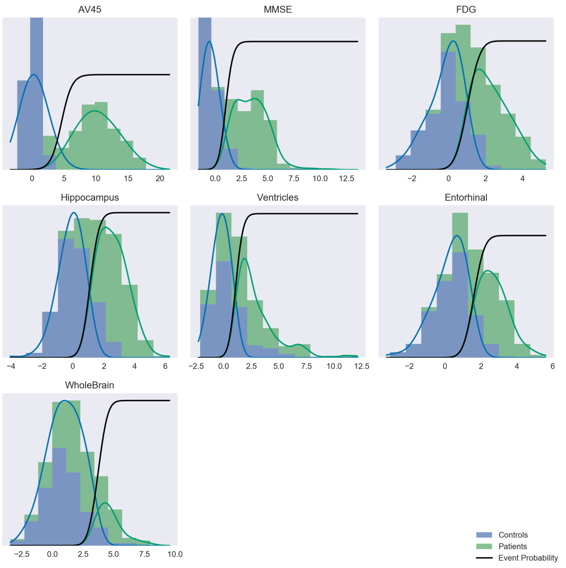

(a) EBM00: 106 controls, 122 patients.

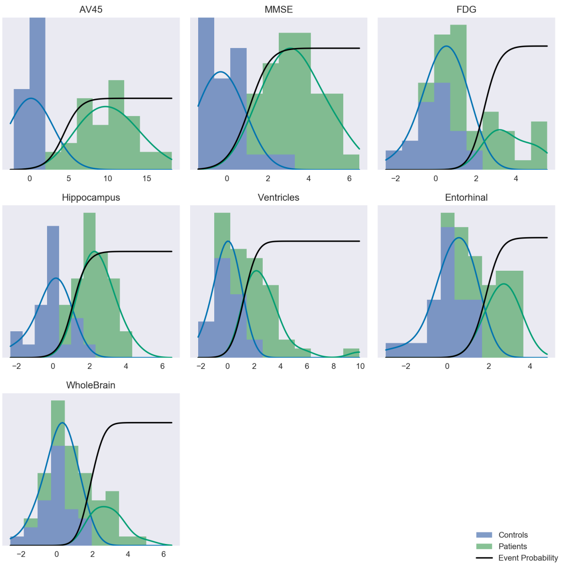

(b) EBM0: 26 controls, 38 patients.

Figure S1: Biomarker Event Measures (EBM00 and EBM0): normalised histograms, Kernel Density Estimate mixture-model components, and event probabilities for the non-network biomarkers included in (a) EBM00 (data from ADNIGO/2) and (b) EBM0 (data from *The168*, a subset of ADNIGO/2). Horizontal axis is c-score (z-score relative to controls).

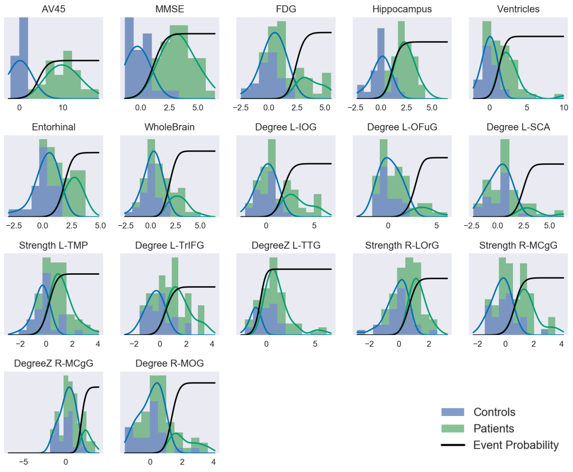

(a) EBM1: hub biomarkers in *The168*.

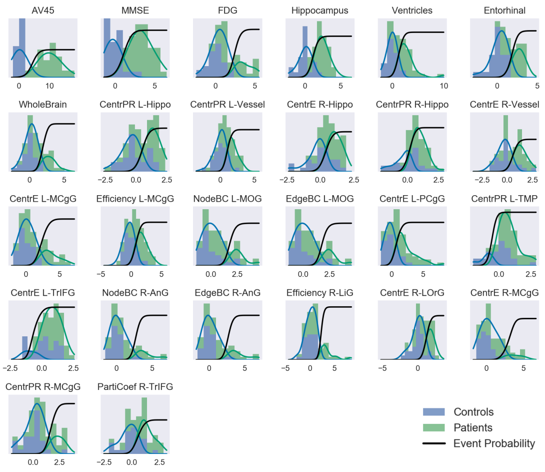

(b) EBM2: centrality biomarkers in *The168*.

Figure S2: Biomarker Event Measures (EBM1, EBM2, EBM4): normalised histograms, Kernel Density Estimate mixture-model components, and event probabilities for biomarkers included in (a) EBM1 (non-network + hub markers) and (b) EBM2 (non-network + centrality markers). EBM4 contains the union of these biomarkers. Abbreviations explained in the main article. Horizontal axis is c-score (z-score relative to controls).
